# Potential Impact of Antiretroviral Therapy and Screening on Cervical Cancer Mortality in HIV-Positive Women in Sub-Saharan Africa: A Simulation

**DOI:** 10.1371/journal.pone.0018527

**Published:** 2011-04-04

**Authors:** Julius Atashili, Jennifer S. Smith, Adaora A. Adimora, Joseph Eron, William C. Miller, Evan Myers

**Affiliations:** 1 Faculty of Health Sciences, University of Buea, Buea, Cameroon; 2 Gillings School of Global Public Health, University of North Carolina at Chapel Hill, Chapel Hill, North Carolina, United States of America; 3 Duke Medical School, Duke University, Durham, North Carolina, United States of America; Instituto de Pesquisa Clínica Evandro Chagas/Fundação Oswaldo Cruz, Brazil

## Abstract

**Background:**

Despite having high cervical cancer incidence and mortality rates, screening for cervical precancerous lesions remains infrequent in sub-Saharan Africa. The need to screen HIV-positive women because of the higher prevalence and faster progression of cervical precancerous lesions may be heightened by the increased access to highly-active antiretroviral therapy (HAART). Policymakers need quantitative data on the effect of HAART and screening to better allocate limited resources. Our aim was to quantify the potential effect of these interventions on cervical cancer mortality.

**Methods and Findings:**

We constructed a Markov state-transition model of a cohort of HIV-positive women in Cameroon. Published data on the prevalence, progression and regression of lesions as well as mortality rates from HIV, cervical cancer and other causes were incorporated into the model. We examined the potential impact, on cumulative cervical cancer mortality, of four possible scenarios: no HAART and no screening (NHNS), HAART and no screening (HNS), HAART and screening once on HAART initiation (HSHI), and HAART and screening once at age 35 (HS35). Our model projected that, compared to NHNS, lifetime cumulative cervical cancer mortality approximately doubled with HNS. It will require 262 women being screened at HAART initiation to prevent one cervical cancer death amongst women on HAART. The magnitudes of these effects were most sensitive to the rate of progression of precancerous lesions.

**Conclusions:**

Screening, even when done once, has the potential of reducing cervical cancer mortality among HIV-positive women in Africa. The most feasible and cost-effective screening strategy needs to be determined in each setting.

## Introduction

Cervical cancer is one of the leading causes of cancer death among women in sub-Saharan Africa [1]. More than 10 million HIV-infected women also live in this region [2]. Compared to HIV-negative women, HIV-positive women have a higher prevalence of cervical precancerous lesions as well as a faster progression of these lesions to invasive cancer [3]–[7].

Mortality from cervical cancer and mortality from other HIV-associated diseases can be competing risks in the evolution of the other disease. Death from cervical cancer would prevent further progression of HIV disease and, prior to the advent of highly active antiretroviral therapy (HAART), the progression of cervical precancerous lesions to cancer was largely averted by early death from AIDS and other opportunistic infections. We hypothesize that the increased survival that is expected to result from increased access to HAART may be large enough to allow for lesions to progress to invasive cervical cancer and thus is likely to be followed by an increase in cervical cancer incidence and mortality.

In developed countries, the potential increase in cervical cancer mortality in HIV-positive women is curtailed by systematic and frequent screening in these women [8]. Despite having a higher HIV prevalence and cervical cancer incidence, screening remains very infrequent in developing countries presumably because of resource-limitations. Policymakers need data on the effect of screening on cervical cancer mortality to better allocate limited resources.

The long term effect of cervical cancer screening in HIV-positive women in sub-Saharan Africa, particularly in the era of HAART remains unknown. Although HAART is expected to increase cervical cancer mortality while screening is expected to reduce this mortality, the magnitude of these effects need to be estimated to better guide policy. In this paper, we estimate the size the potential effect of HAART therapy with or without screening on the mortality due to cervical cancer in Cameroon.

## Methods

### Model structure

**Figure 1:**
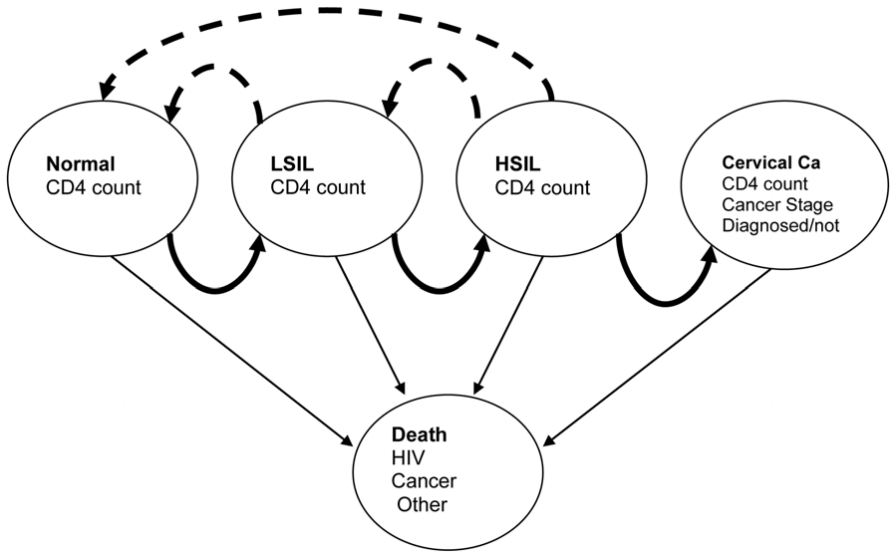
Summary of states in the Markov model. Adapted from an original depiction by Goldie et al, 1999 [10].

We developed a state-transition Markov model, using TreeAge Pro™ 2008 Healthcare Module (TreeAge Software Inc., Williamstown, MA, USA). This type of model allows analysts to model transitions of a cohort of patients in a number of health states over a long period of time subdivided into a series of short intervals [9]. Our model was designed to simulate the evolution over time of HIV infection and cervical precancerous and cancerous lesions in a cohort of HIV-positive women in Cameroon. The primary structure of the Markov model was based on a previous description of a model implemented by Goldie et al, 1999 [10] in HIV-positive women in the US (Figure 1). In brief, the model summarizes the progression of cervical neoplasia in HIV in five states: normal with no lesion, low-grade squamous intra-epithelial lesions (LSIL), high-grade squamous intra-epithelial lesions (HSIL), invasive cervical cancer and death. Each of the four (non-death) states is stratified by CD4 cell count. The cancer stage is further stratified by stage of cervical cancer and whether cancer has been diagnosed (thus being treated) or not. During their lifetime, women's disease state can progress from normal to LSIL to HSIL to cervical cancer. Women in the HSIL and LSIL states can regress to lower states. Death can occur to women in any of the four states and can result from cervical cancer, HIV related-causes or other causes of death.

### Model parameters

Parameters used in the model were abstracted from the published literature and reflected data for Cameroon as much as possible. The values used in the baseline model are shown in Table 1. The base model was designed to simulate the progression over time of a cohort of HIV-positive women aged 25 with CD4 count >500, 30% of whom had precancerous lesions (one-third of which were high grade lesions) [11]. All cause age-specific mortality rates were estimated based on abridged life tables for women in Cameroon [12]. HIV mortality rates were estimated based on WHO data [13].The proportion of HIV-mortality that occurs in each CD4 category was estimated based on data by Goldie et al [10]. Cervical cancer mortality rates were also abstracted from WHO estimates of annual cervical cancer incidence and deaths in Cameroon [1]. Age-specific mortality rates from other causes were estimated by adjusting (using another Markov analysis) all-cause age-specific mortality rates to deduct mortality from cervical cancer and mortality from HIV.

**Table 1:** Baseline parameters used in modeling cervical cancer mortality in HIV positive women.

Variable	CD4 >500	CD4200–500	CD4<200	Source(reference)
**Initial prevalence of lesions, %**	30	NA	NA	[11]
**Initial proportion of lesions that were HSIL (%)**	33	NA	NA	[11]
**HIV infection**				
**HIV mortality rate (per 1000 per year)** *	0.05	6.06	48.6	[13]
**Effect of HAART in reducing HIV mortality**	NA	NA	4-fold	[14], [15]
**HIV progression rate (per 100 per year)** **	18.1	27.5	NA	[10]
**Cervical Cancer**				
**Cancer mortality rate (per 1000 per year)** *				[1]
**Local invasive cancer**	41.1	41.1	41.1	
**Regional invasive cancer**	222.1	222.1	222.1	
**Distant invasive cancer**	543.5	543.5	543.5	
**Progression rate (per 100 per year)**				[10]
**Normal to LSIL**	0.016	0.67	0.67	
**LSIL to HSIL**	0.73	2.93	2.93	
**HSIL to local invasive cancer**	2.0	2.42	2.42	
**Local to regional invasive cancer**	4.03	4.03	4.03	
**Regional to distant invasive cancer**	4.03	4.03	4.03	
**Regression rate (per 100 per year)**				[10]
**LSIL to normal**	2.99	2.99	2.99	
**HSIL to LSIL**	0.30	0.30	0.30	
**HSIL to normal**	0.30	0.30	0.30	
**Screening test**				[10]
**Sensitivity, %**	70	70	70	
**Specificity, %**	90	90	90	

*Mortality in each CD4 category or cervical cancer stage were determined by weighting crude estimates by the proportions due to each category or stage from Goldie et al,1999 [10].

**estimated from mean duration at each stage.

In the absence of published data on long-term progression or regression rates of precancerous lesions in HIV positive women in Cameroon, we used published estimates from women in the pre-HAART era from Goldie et al [10]. The effect of HAART on HIV/AIDS-related mortality was also estimated based on the published literature [14], [15].

### Model assumptions

Key simplifying assumptions of the model include: 1) The natural history of cervical cancer involves progress from normal to LSIL to HSIL to local cancer to regional/distant cancer to death from cancer, without skipping. 2) The regression of neoplasia can only be from HSIL to normal or LSIL, of from LSIL to normal. A patient cannot regress from cancer. Cancer stages also cannot regress. 3) HIV diseases progression is only from CD4 500+ to 200–500 to <200. This is a historic parameter, indicating the advance in HIV disease. Once a patient has CD4 <200 she will always be classified in the CD4 <200 category, even if her actual CD4 count improved with treatment. In other words worsening HIV-disease cannot regress. This assumption is consistent with data that show that improvements in antiretroviral therapy do not appear to reduce the progression of precancerous lesions even amongst women with improved CD4 counts [16]. 4) The progression/regression rate of cervical precancerous lesions is not dependent on a previous history of precancerous lesions as the parameters used in the model are from heterogeneous populations (that include patients with and without a previous history of lesions) [17].

### Scenarios and outcomes assessed

We assessed the projected lifetime cumulative mortality due to cervical cancer in four plausible scenarios of HIV and cervical cancer care in Cameroon: no HAART and no screening (NHNS), HAART when indicated and no screening (HNS), HAART when indicated and screening on HAART initiation (HSHI), and HAART when indicated and a single screen at age 35. The age 35 was selected based on WHO recommendations for screening in resource-limited settings [18].

### Sensitivity analyses

The sensitivity of cumulative cervical cancer mortality to parameter estimates was analyzed in one-way sensitivity analyses. We were particularly interested in the sensitivity of HAART effectiveness in lowering HIV-mortality and SIL progression rates since the baseline values used were all external to the study and these values are likely to vary substantially depending on the study population.

## Results

### Base model

A substantial proportion of women were expected to die from cervical cancer. The baseline model projected a lifetime cumulative cervical cancer mortality of 25.4 per 1000 HIV-positive women who were infected at age 25 and neither received HAART nor were screened for cervical cancer (Table 2). Cumulative cervical cancer mortality doubled to 46.6 per 1000 HIV-positive women who were infected at age 25, were placed on HAART when their CD4 went below 200cells/mm3 and had no screening for cervical cancer. If the latter women were screened either once at HAART initiation or once when they were aged 35, then mortality could reduce to 42.8 and 41.7 per 1000 women respectively. Interestingly, cervical cancer mortality following HAART and screening once at HAART initiation was still higher than cervical cancer mortality associated with no HAART and no screening.

**Table 2:** Projected cumulative (lifetime) cervical cancer mortality in HIV-positive women in Cameroon on HAART and or screened for cervical cancer.

Intervention	Mortality per 1000	NNT	NNS
No HAART, No Screen	25.4	Ref.	-
HAART, No Screen	46.6	47	Ref
HAART+ Screen once at HAART initiation	42.8	-	262
HAART+ Screen once at age 35	41.7	-	202

NNT - Number of women who need to receive HAART for each additional cancer death.

NNS - Number of women who need to be screened for each cancer death prevented.

Ref: Referent.

In absolute measures, these mortality projections meant that compared to no HAART and no screening, an additional cervical cancer death would occur for every 47 women put on HAART when indicated, but not screened. Conversely, once women were put on HAART as indicated, then screening once at HAART initiation would prevent one case of cancer for every 262 women screened. Screening once at age 35 was projected to prevent one cancer death per 202 women screened.

**Figure 2:**
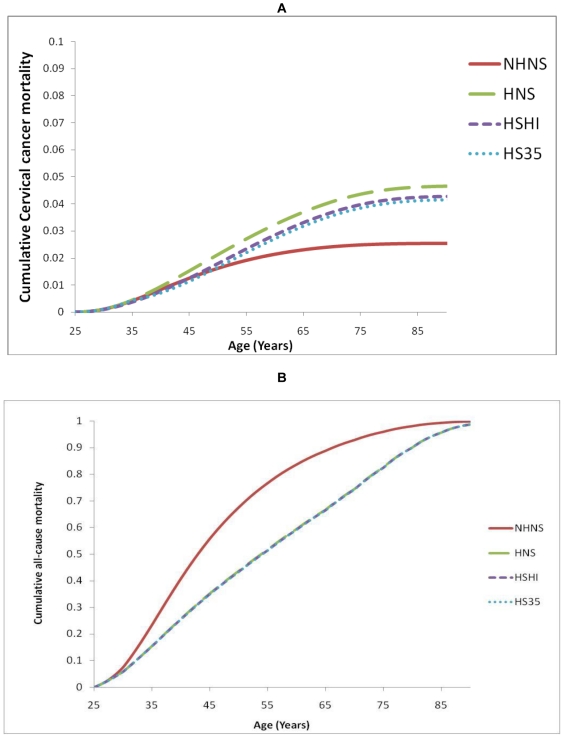
Cumulative mortality by intervention in a cohort of HIV positive women getting infected at age 25. A. Cumulative mortality from cervical cancer. B. Cumulative mortality from all causes (NHNS: No HAART No Screening; HNS: HAART but No Screening; HSHI: HAART and one screen at HAART initiation; HS35: HAART and one screen at age 35).

The timing of cervical cancer deaths was also influenced by the type of intervention received (Figure 2). With NHNS the majority of cervical cancer deaths occurred within the second and third decade after diagnosis. In patients on HAART the occurrence of deaths was further delayed beyond the third decade after infection.

Only HAART had a substantial impact on the overall survival from all causes of death after infection (Figure 2 and Table 3). Compared to no HAART and no screening, all three scenarios with HAART (HAART with no screening, HAART with one screen at HAART initiation and HAART with one screen at age 35) resulted in gains in life-expectancy. However, there was only minimal difference in survival from all causes under the three scenarios with HAART, with one time screening only slightly increasing survival (Table 3).

**Table 3:** Projected cumulative cause of mortality and gains in life expectancy in HIV-positive women in Cameroon on HAART and or screened for cervical cancer.

Intervention	Cause of mortality (proportion)			Gains in life expectancy (years)	
	HIV/AIDS	Cervicalcancer	Other cause	Due to HAART	Due to Screening
No HAART, No Screen	63.3%	2.5%	34.1%	Ref.	-
HAART, No Screen	28.3%	4.7%	66.8%	10.6	Ref.
HAART+ Screen once at HAART initiation	28.4%	4.3%	67.1%	-	0.09
HAART+ Screen at age 35	28.4%	4.2%	67.2%	-	0.11

Ref: Referent.

### Sensitivity analysis

**Figure 3:**
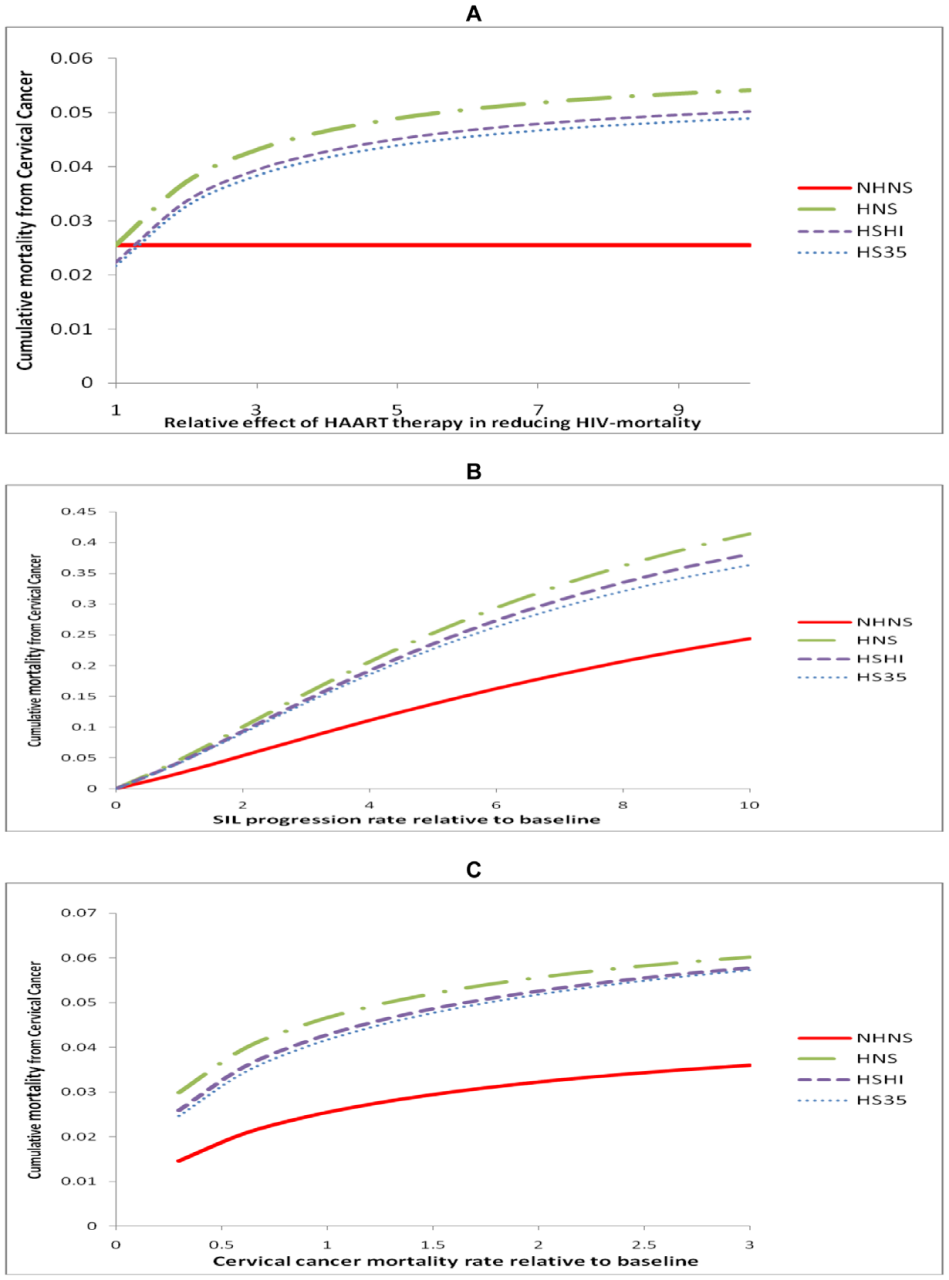
Sensitivity of cumulative cervical cancer mortality to model parameters. A. Sensitivity to the relative effect of HAART in reducing HIV-mortality. B. Sensitivity to the progression rate of precancerous lesions. C. Sensitivity to cervical cancer mortality rate. The values on the x-axis refer to the ratio of each parameter compared to its value in the base model. (NHNS: No HAART No Screening; HNS: HAART but No Screening; HSHI: HAART and one screen at HAART initiation; HS35: HAART and one screen at age 35).

The projected cervical cancer mortality estimates were robust to the magnitude of the effect of HAART in reducing HIV-related mortality (Figure 3). All four mortality estimates were within 10% of their baseline value unless the effect of HAART was below a two-fold decrease in HIV mortality. Further increasing the effect of HAART only slightly increased cumulative cervical cancer mortality.

Mortality estimates were most sensitive to the rate of progression of precancerous lesions to more severe lesions or invasive cancer (Figure 3). In all four scenarios cumulative mortality increased with faster progression rates. Doubling the progression rate resulted in a near doubling of cervical cancer mortality. Nevertheless, the relative mortality between each of the scenarios remained constant across progression rates.

**Figure 4:**
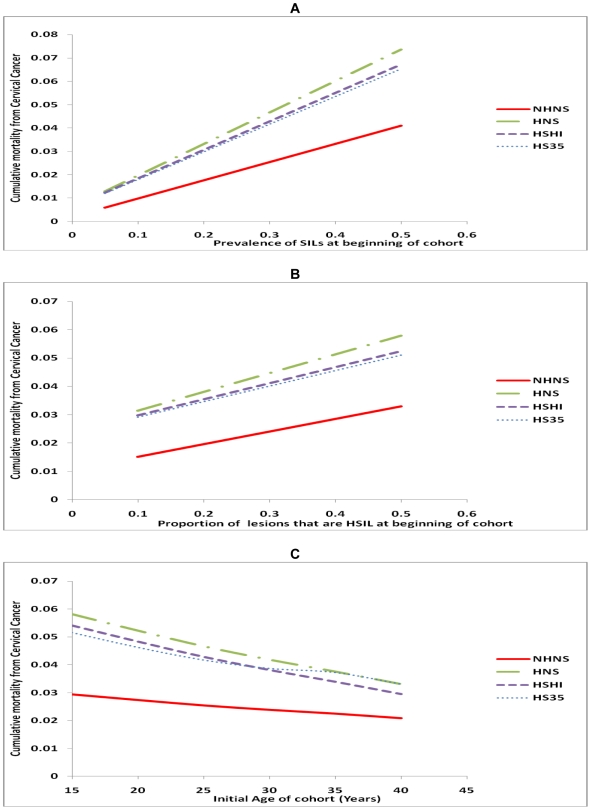
Sensitivity of cumulative cervical cancer mortality to baseline parameters. A. Sensitivity to the prevalence of squamous intraepithelial lesions at beginning of cohort. B. Sensitivity to the proportion of lesions that are high grade squamous intraepithelial lesions at the beginning of cohort. C. Sensitivity to the initial age of cohort. (NHNS: No HAART No Screening; HNS: HAART but No Screening; HSHI: HAART and one screen at HAART initiation; HS35: HAART and one screen at age 35).

In contrast to the sensitivity of the model to the progression rate of lesions, the model mortality estimates were rather robust to other parameters such as cancer mortality rates and other baseline parameters. The cumulative cervical cancer mortality only slightly increased with increases in cervical cancer mortality rates (Figure 3). Cumulative cervical cancer mortality was also robust to the baseline prevalence of lesions (Figure 4) and the proportion of lesions that were high grade lesions (Figure 4). As expected, mortality from cervical cancer decreased as the age of HIV infection (the initial age) of the cohort increased (Figure 4). Screening test sensitivity had very little impact on cervical cancer mortality with mortality only slightly decreasing as sensitivity increased. Life expectancy also increased only slightly with improved sensitivity - a gain of 0.06 years as sensitivity increased from 50% to 100%. Screening test specificity had no impact on cervical cancer mortality or life expectancy.

## Discussion

The magnitude and the impact of HAART treatment and screening, on cervical cancer mortality in HIV-positive women in sub-Saharan Africa remain unknown. The ethical and practical complexities of potentially denying care to patients and following women for their lifetime respectively make it unfeasible to conduct a study to quantify this impact. Computer-based simulation models, however, provide an alternative means of quantifying the potential impact of these interventions. These models are of even greater importance in sub-Saharan Africa, where real-life estimation is hampered by limitations in the long-term follow-up of patients, in cancer diagnosis and in the determination of cause of death.

In this paper, we used a mathematical model to project that mortality due to cervical cancer in HIV positive women in Africa is potentially very high. While this mortality can be worsened by providing HAART without screening, screening can be associated with non-negligible reductions in mortality from cervical cancer. These data confirm and quantify the potential gains of cervical cancer screening in HIV-positive women in regions with high cancer incidence and mortality such as Cameroon. With an estimated 200,000 HIV-infected women in Cameroon [12], our projections imply that screening these women once at HAART initiation would prevent close to 763 deaths due to cervical cancer, while screening these women once at age 35 could prevent approximately 990 deaths in these women.

WHO guidelines for screening in resource-limited settings include at least a one-time screening in the third or fourth decade of life [17]. Our projected potential gains with one-time screening in HIV-positive women are much smaller than those in other settings where screening is systematic and more frequent. For example, screening in the UK was estimated to prevent one death for every 65 women screened systematically [19]. The reductions in cervical cancer mortality associated with a single screen in HIV-positive women were also less compared to the estimated 25–36% reductions in lifetime risk reported in models of HIV-negative women in five other developing countries [20]. These differences could be due to a higher incidence and faster progression of precancerous lesions in HIV-positive women compared to HIV-negative women [7].

Although we show potential benefits of screening, our analysis did not take the cost of screening into account. A formal assessment of the cost-effectiveness of screening in this and other settings will provide additional information regarding resource needs and potential effects for cervical cancer screening. Our data show that even one time screening at HAART initiation or at age 35 would potentially be beneficial. According to guidelines from the Commission on Macroeconomics and Health, a policy is generally considered cost-effective if the incremental cost per life saved is less than the country's GDP per capita [21]. Our analyses estimate that one-time screen policies would improve life expectancy by approximately 0.10 years. With Cameroon's GDP per capita being estimated at USD 1,019 [22], we further estimated that a one-time screening strategy will need to cost at most 101 USD per screen for it to be considered cost-effective. This cost estimates includes both direct costs (such as cost of the test) and indirect costs (such as costs of running the screening program). More formal cost-effectiveness analyses including appropriate weighting for disability and discounting will be needed to confirm this.

As with every simulation exercise, the model form, parameters and assumptions present potential limitations to the confidence with which inferences from the analysis can be made to real-life scenarios. We choose a model that had previously been validated in the immediate pre-HAART era, a population similar to our target in this analysis. However some of the model parameters which could not be directly estimated were estimated indirectly using either US or WHO data. In the absence of prospective data on cervical cancer progression and regression rates for Cameroonian women, we used published data from the pre-HAART era in the US and assessed the impact of this choice in sensitivity analyses. A faster progression rate in developing countries could translate to even higher mortality due to cervical cancer. Nevertheless, the relative effects of HAART and screening were projected to remain similar, while the number of deaths averted with screening would increase as progression rates increase. On the other hand, if HAART was shown to have an effect on the progression of lesions, a finding that has been inconsistent [15], then the mortality due to cancer as well as the gains associated with screening would be reduced.

A further limitation, by design, of this analysis is the absence of any consideration for HPV infection status and/or HPV vaccines. The former was due to the absence of reliable data on HPV and the progression of lesions in HIV in sub-Saharan Africa. It is thus difficult to infer on the potential impact of an HPV test. We however suspect that one-time screening with an HPV-test could be better than a one-time cytology screen, as the HPV test will have a higher sensitivity, be able to detect lesions at earlier stages (allowing for early treatment) and thus result in lower mortality. Indeed, a recent study in population (of mainly HIV-negative women) in rural India reported a 50% reduction in cervical cancer mortality (measured as hazards of mortality) following a one-time HPV test [23]. They also report a low insignificant reduction in mortality with cytology and VIA. These findings however need to be verified in a population of HIV-positive women, in whom the greater variability of HPV types, higher HPV-persistence rates and faster progression of lesions could mean reduced effect of an HPV test used for one-time screening.

While cervical cancer screening could substantially reduce mortality due to cervical cancer, it was projected to have very little effect on all cause mortality. We did not assess the quality of life gains, but these data indicate that even with screening further gains in overall life expectancy will depend on the extent of prevention and care for other causes of mortality including opportunistic infections.

In conclusion, cervical cancer could account for a high proportion of deaths among HIV- positive women in Africa once they have access to HAART. These deaths could be reduced with screening, even when done just once. Antiretroviral treatment scale-up activities need to be followed by strategies to systematically increase access to screening services as well as treatment for cervical precancerous and cancerous lesions as needed. While the feasibility and cost-effectiveness of more frequent screening is still to be assessed, women need to be provided the opportunity to get screened at least once on initiating HAART or at age 35.
